# Prevalence of depression among breast cancer patients in Asia: a systematic review and meta-analysis

**DOI:** 10.3389/fonc.2026.1665569

**Published:** 2026-03-03

**Authors:** Wanqing Mo, Xiufang Ding, Xingxing Wu

**Affiliations:** Department of Breast, Huzhou Maternity and Child Health Care Hospital, Huzhou, Zhejiang, China

**Keywords:** Asia, breast cancer, depression, meta-analysis, prevalence

## Abstract

**Background:**

While breast cancer (BC) patients face a heightened risk of depression, regional variations in prevalence within Asia remain underexplored. This study aimed to systematically review and meta-analyze the prevalence of depression among BC patients in Asia, and to explore variations by region and treatment modality.

**Methods:**

We searched multiple databases (e.g., PubMed, Embase, CNKI) for studies reporting depression prevalence in Asian adult BC patients up to May 2025. Inclusion required the use of standardized diagnostic or validated screening tools. Data extraction and quality assessment followed standard systematic review procedures. Pooled prevalence estimates were derived using a random-effects model in R software.

**Results:**

The meta-analysis revealed that the pooled prevalence of depression among Asian breast cancer patients was 28% (95% CI: 0.20–0.36). However, subgroup analyses were inconclusive regarding differences by region or treatment, likely due to limited representation within each subgroup.

**Conclusions:**

The findings suggest a high incidence of depression in breast cancer patients, underscoring the importance of integrating psychological interventions into their treatment plans. However, as analyses for regional or treatment-related differences were inconclusive, future research—particularly with larger samples from diverse settings—is needed to clarify these associations and better inform the development of tailored intervention strategies for BC patients across diverse Asian regions.

## Introduction

Breast cancer (BC) is a prevalent malignancy among women globally, with significant contributions to cancer-related morbidity and mortality (1). Beyond the physical challenges posed by the disease, BC patients often encounter severe psychological difficulties, with depression being a prominent concern (2, 3). The presence of depression in BC patients can negatively influence treatment adherence, quality of life, and overall prognosis (4).

The prevalence of depression among BC patients can be affected by a variety of factors, including demographic variables, clinical factors, and psychosocial elements. Demographic factors such as age, marital status, and socio-economic status play a crucial role, with younger patients and those lacking social support being particularly vulnerable (5, 6). Clinical aspects, including cancer stage at diagnosis, type of treatment received (e.g., chemotherapy, surgery, radiation therapy), and comorbid conditions, also significantly affect the likelihood of depression (7, 8). Moreover, psychosocial factors such as social support levels, prior mental health history, and coping mechanisms are vital determinants of mental health outcomes in these patients (9).

Despite the recognition of these influencing factors, a significant variation in the reported prevalence of depression among breast cancer patients persists across studies in Asia. Among the included Asian studies, prevalence ranged from approximately 3.71% in a Korean national cohort to 51.02% in a Jordanian study, with other countries also reporting high rates (e.g., 48% in China, 44.51% in Syria, 39.53% in Vietnam), suggesting that regional and methodological differences may jointly drive the wide dispersion of estimates (10–14). This variability may stem from differences in study design, sample characteristics, and methodological approaches to diagnosing depression (15, 16).

Breast cancer patients often face a significant psychological burden throughout diagnosis, treatment, and follow-up. Depression not only affects quality of life and treatment adherence but may also increase healthcare costs and hinder long-term recovery. Previous systematic reviews and meta-analyses indicate a considerable global pooled prevalence of depression in breast cancer patients, at approximately 30%–32% (17). Furthermore, the burden of depression shows marked regional variation. When stratified by WHO region, prevalence is approximately 49%–51% in the Eastern Mediterranean and 23%–33% in Southeast Asia (18), suggesting associations with socioeconomic development, healthcare accessibility, and cultural context.

Consequently, we systematically reviewed the existing literature on depression among breast cancer patients in Asia and conducted this meta-analysis. Our aims are to investigate regional variations and understand how various factors contribute to these differences. This comprehensive synthesis will help inform the development of targeted mental health interventions to better support breast cancer patients in Asia.

## Methods

### Search strategy

A systematic literature search was conducted in the following databases: the English databases PubMed, Wiley Library, Embase, Cochrane Library, and PsycINFO, as well as the Chinese database CNKI. The search terms included “breast cancer” OR “breast neoplasm”, “depression” OR “depressive disorder”, “prevalence” OR “epidemiology” and their combinations, using Boolean operators (AND/OR). The search period covered from the inception of each database to May 31, 2025, and the search was restricted to publications in English and Chinese. Although platforms containing Chinese academic literature were searched, the inclusion criteria for this systematic review were limited to studies published in full text in English or Chinese. Due to constraints in resources and translation capacity, we did not attempt to translate or include studies published in other Asian languages (e.g., Korean, Japanese).

### Inclusion and exclusion criteria

#### Inclusion criteria

1. Subjects: Female patients diagnosed with breast cancer, with no restrictions on age or nationality. Studies must include patients assessed for depression using standardized diagnostic criteria, such as the Diagnostic and Statistical Manual of Mental Disorders (DSM) or the International Classification of Diseases (ICD), or validated screening tools such as the Hospital Anxiety and Depression Scale (HADS) or the Beck Depression Inventory (BDI).
2. Geographical Scope: Studies conducted in Asian countries, including but not limited to China, India, Japan, South Korea, Malaysia, Palestine, Vietnam and Thailand.
3. Type of Study: Randomized controlled trials, cohort studies, case-control studies, and cross-sectional studies that investigate the prevalence of depression among breast cancer patients.
4. Outcome Measures: Studies that report the prevalence of depression or provide data on the incidence of depression among breast cancer patients.
5. Study Quality: Research published in peer-reviewed journals.

#### Exclusion criteria

1. Incomplete Data: Studies with incomplete original data that do not allow for extraction of relevant information regarding depression risk factors and outcomes.
2. Duplicate Publications: Repeatedly published literature or duplicate studies to ensure that each study is only included once.
3. Non-Compliant Interventions: Studies that do not adhere to standardized diagnostic criteria or validated screening tools for assessing depression, or studies that focus solely on treatment outcomes without evaluating risk factors.
4. Lower quality: Studies published in non-peer-reviewed journals.

### Data extraction

The literature screening and data extraction were strictly conducted independently by two researchers according to the inclusion and exclusion criteria, followed by cross-checking. In cases of disagreement, a third researcher was consulted to resolve the issue through discussion. The extracted data included the author, year, study type, sample size, age, identified risk factors, and outcome measures related to depression.

### Literature quality assessment

The literature included in this study was retrospectively analyzed using the JBI Critical Appraisal Checklist (JBI). The studies were categorized based on their evaluation as follows: high quality: score ≥80%; moderate quality: score between 50% and 79%; low quality: score <50%.

### GRADE evidence evaluating system

The quality of evidence was assessed using GRADE profiler 3.6 (19), categorizing it into high, moderate, low, and very low levels. The assessment criteria included risk of bias, inconsistency, indirectness, imprecision, and publication bias.

### Statistical methods

A meta-analysis of the point prevalence of depression among breast cancer patients in Asia was performed using R software. The outcome was defined as the depression prevalence reported at a specific assessment time point in each study. In anticipation of substantial clinical and methodological heterogeneity, a random-effects model was employed for pooling, and the restricted maximum likelihood (REML) method was used to estimate the between-study variance (τ²). Proportion data from individual studies were pooled after Freeman-Tukey double arcsine transformation to stabilize variances and handle extreme proportions. The results are reported as the pooled prevalence (PP) with its 95% confidence interval (95% CI) on the original scale after back-transformation. Heterogeneity was quantified using the I² statistic, τ², and Cochran’s Q test (with reported p-value). All results are presented visually in a forest plot.

To explore sources of heterogeneity, subgroup analyses were further conducted by geographical region and treatment type (chemotherapy, surgery, or both). Differences between subgroups were tested for statistical significance using the Q-test based on meta-regression. The following sensitivity analyses were performed to examine the robustness of the results: leave-one-out analysis, subgroup analysis by depression assessment tool/cut-off, and subgroup analysis by risk of bias. Potential small-study effects (publication bias) were assessed using a funnel plot and Egger’s regression test.

## Results

**Figure 1:**
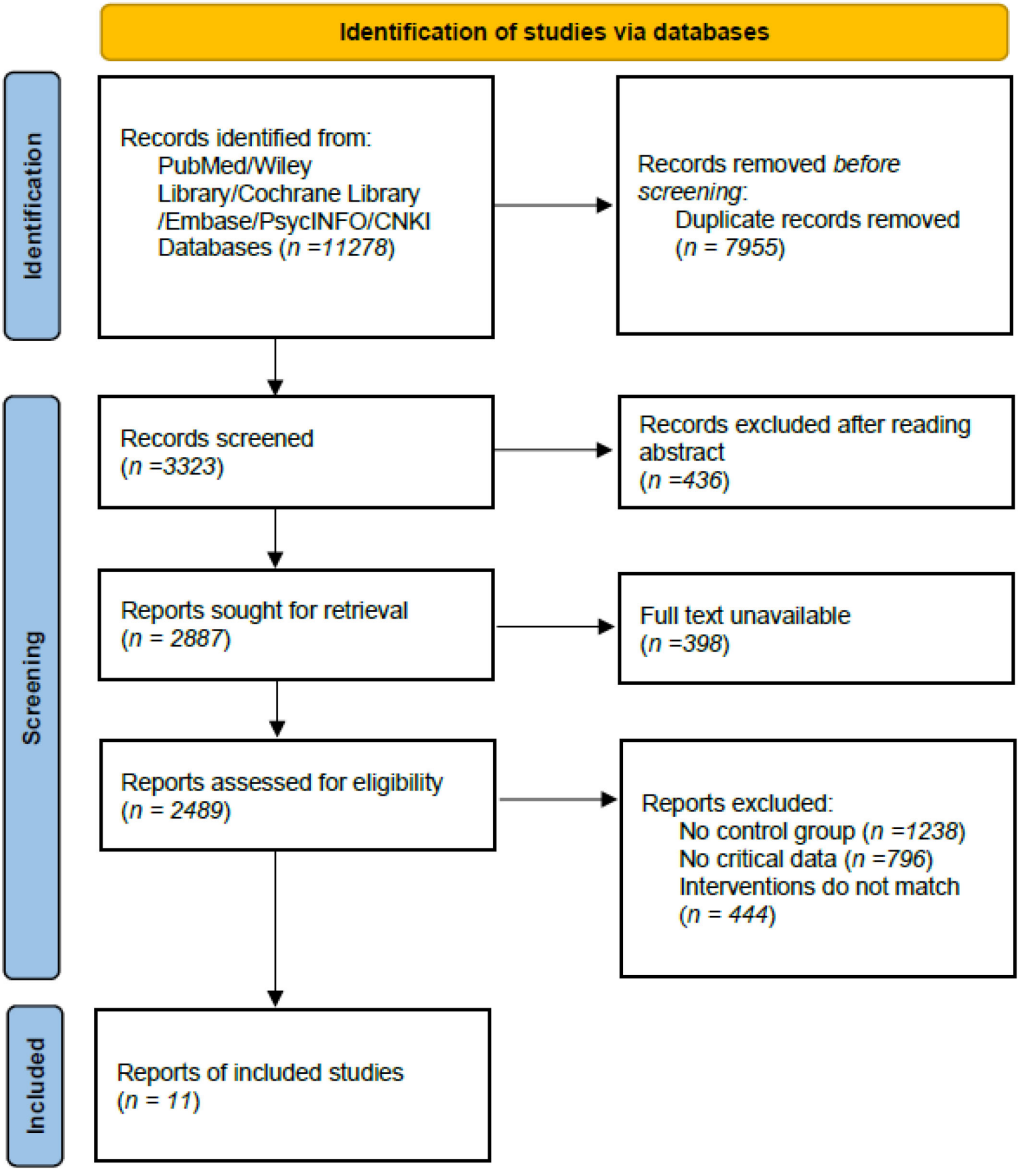
Literature screening flowchart.

From the online databases, a total of 11,278 records were retrieved. After removing 7,955 duplicates and excluding 436 records based on title/abstract screening, 2,887 reports remained for full-text screening. Of these, 11 articles met the inclusion criteria, and one additional study was identified through citation searching, resulting in 12 studies included in the quantitative synthesis. The PRISMA flow chart diagram is presented in **Figure 1**.

### Study characteristics

All 11 high quality studies, assessed by the JBI quality assessment checklist (20), are included in this meta-analysis and are summarized in **Table 1**. A total of 89,615 cases were reported spanning South Korea (10), Oman (21), China (12, 22), Malaysia (23), Iran (24, 25), Palestine (26), Jordan (11), Syria (13) and Vietnam (14). Most studies employed a cross-sectional study design, with three being cohort studies, and the majority had a sample size between 160 and 300. Two studies used the HADS scale (21, 23) to assess outcomes, three studies used the PHQ scale (12, 13, 26), two studies used the ZDSS scale (22, 25), and the remaining studies used the BDI-II (24), DASS (11), EQ-5D-5L (14), and ICD-9 (10) criteria, respectively. These studies can also be subgrouped based on the breast cancer treatment received by participants: four studies included patients receiving chemotherapy only (12, 14, 24, 26), three studies included patients undergoing surgery only (10, 11, 26), two studies included patients receiving both chemotherapy and surgery (21, 22), and the remaining studies included patients who were diagnosed but had not yet started treatment (13, 23, 25).

**Table 1:** Characteristics of included studies on depression among breast cancer patients in Asia.

Author	Publication year	Country	Total population analyzed	Age (years)	Outcome measurement	Developed outcome	Prevalence (%)	Study design	Type of study participants	Level of quality	Timing of depression assessment
Heo et al. (10)	2017	South Korea	87842	10+	ICD-9	3256	3.71	Cohort	invasive breast cancer patient underwent surgery	High	Depression ICD codes captured from 1 year before BC diagnosis to at least 6 months after diagnosis/surgery.
Al-Fahdi et al. (21)	2023	Oman	171	18+	HADS	29	16.95	Cross-sectional	BC patients	High	Single timepoint survey during clinic appointment (outpatient visit).
Li (12)	2022	China	300	18+	PHQ-9 & GAD7	144	48	longitudinal prospective study	BC patients	High	Baseline at discharge; follow-ups at 6, 12, and 18 months after discharge.
Hassan (23)	2015	Malaysia	205	20+	HADS	45	21.95	Cross-sectional	BC patients	High	Single timepoint survey during hospital visit.
Shorofi (24)	2021	Iran	120	18+	BDI-II & PSQI	17	14.17	Cross-sectional	non-metastatic unilateral breast cancer undergoing chemotherapy	High	During active chemotherapy (chemotherapy center); mean ~5.52 months after mastectomy.
Sadaqa (26)	2022	Palestine	223	30-70	PHQ-9	70	31.39	Cross-sectional	BC patients	High	Within 1 year after breast cancer diagnosis.
Zhou (22)	2011	China	120	25-65	ZSDS	36	30	Clinical trial	Breast Cancer Patients after Radical Mastectomy	High	Multiple timepoints: 1 day pre-operation; 1 day before discharge; during 2nd and 3rd chemotherapy admissions.
Farbood (25)	2021	Iran	120	25-65	ZSDS	17	14.17	Cross-sectional	breast cancer patient scheduled for surgery	High	Assessed before scheduled surgery (pre-surgery admission).
Obeidat (11)	2020	Jordan	49	23-71	DASS	25	51.02	descriptive correlational survey	unilateral early-stage breast cancer (Stages I–II)	High	–
Soqia (13)	2022	Syria	164	25+	PHQ-2&GAD-2	73	44.51	Cross-sectional study	BC patients	High	Survey administered in the anticancer drug administration room at hospital.
Luu (14)	2024	Vietnam	301	18+	EQ-5D-5L	119	39.53	Cross-sectional study	HER2-positive breast cancer patients	High	Participants had received >=3 treatment cycles before the interview.

### Prevalence of depression among breast cancer patients in Asia

**Figure 2:**
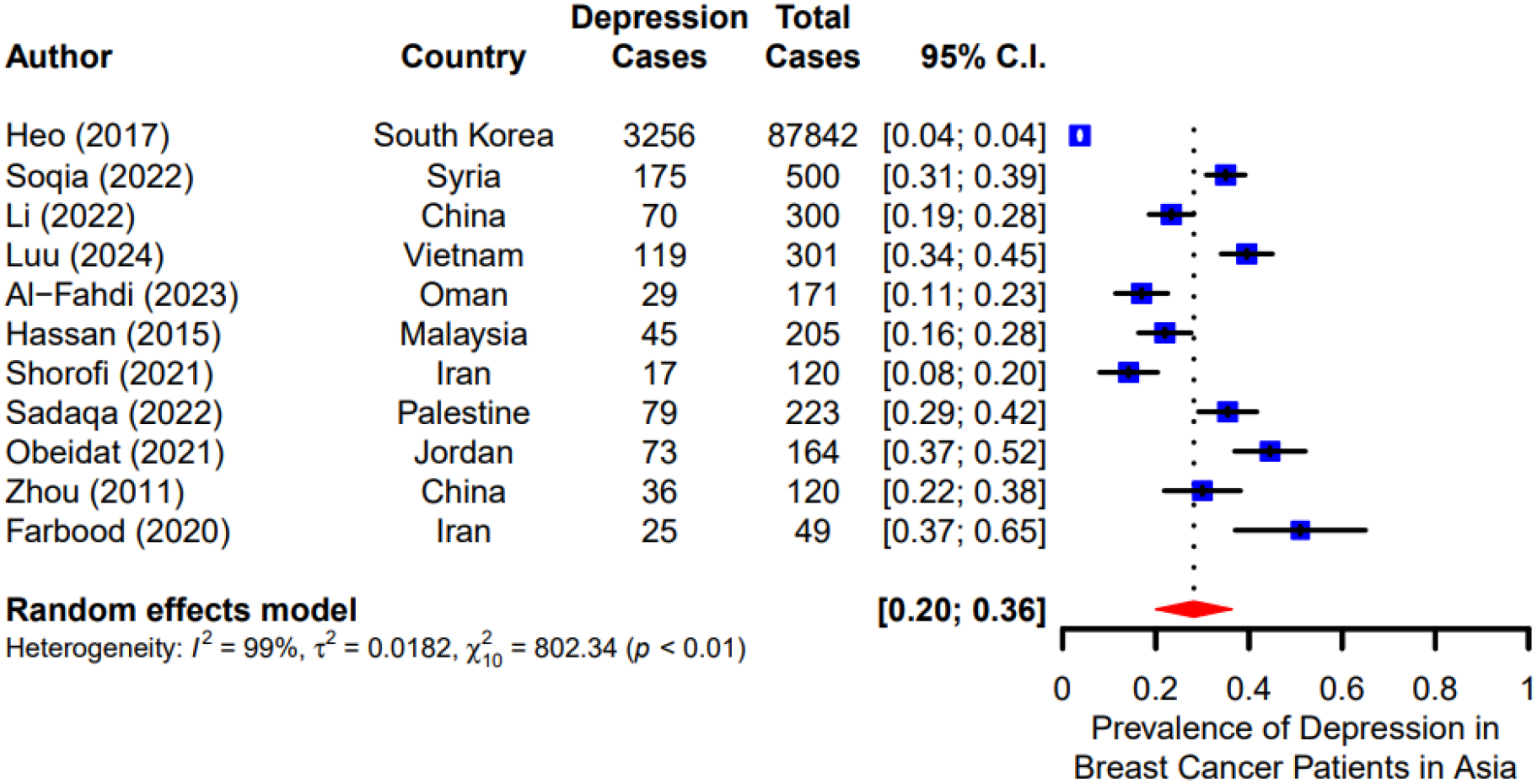
Prevalence of depression among breast cancer patients in Asia.

Due to significant heterogeneity (I² = 99%, p < 0.01), a random-effects model was employed to calculate the overall prevalence. The results indicated that the pooled prevalence of depression among breast cancer patients in Asia was 28% (95% CI: 0.20–0.36) (**Figure 2**).

### Subgroup analysis

**Figure 3:**
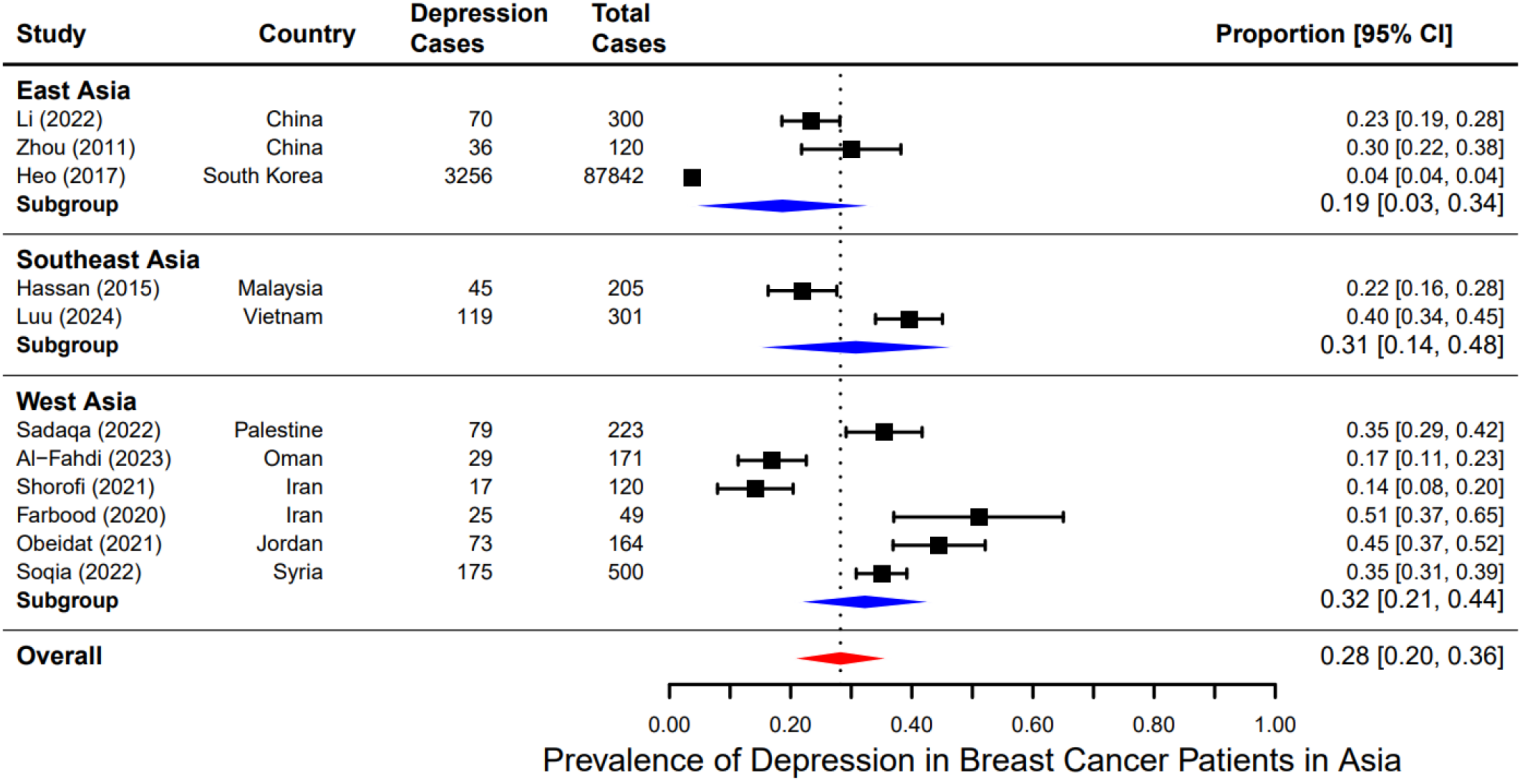
Subgroup analysis of pooled prevalence of depression among breast cancer patients by region.

After confirming a significant rate of depression in Asian BC patients, we then moved on to explore potential factors that may influence these rates, namely differences in geography and treatment type. We first divided all cases into three regions: West, East, and Southeast Asia. The prevalence of depression among East Asian BC patients was slightly lower 19% (95% CI: 3%–34%) (10, 12, 22) than the other two groups: 31% (95% CI: 14%–48%) (14, 23) for Southeast Asia, and 32% (95% CI: 21%–44%) for West Asia (11, 13, 21, 24–26) (**Figure 3**). However, this difference was only trending and did not reach significance (p > 0.05) likely due to the large variability of the reported prevalence of depression. The study from South Korea (12) reported an exceptionally low prevalence of 3.71% (95% CI: 3.58%-3.83%), while Chinese ones (12, 22) reported much higher rates (23% and 30%). This heterogeneity could be due to differences in cultural attitudes towards mental health (27–29), access to healthcare (30–32) and socio-economic factors (18, 29) in these regions.

**Figure 4:**
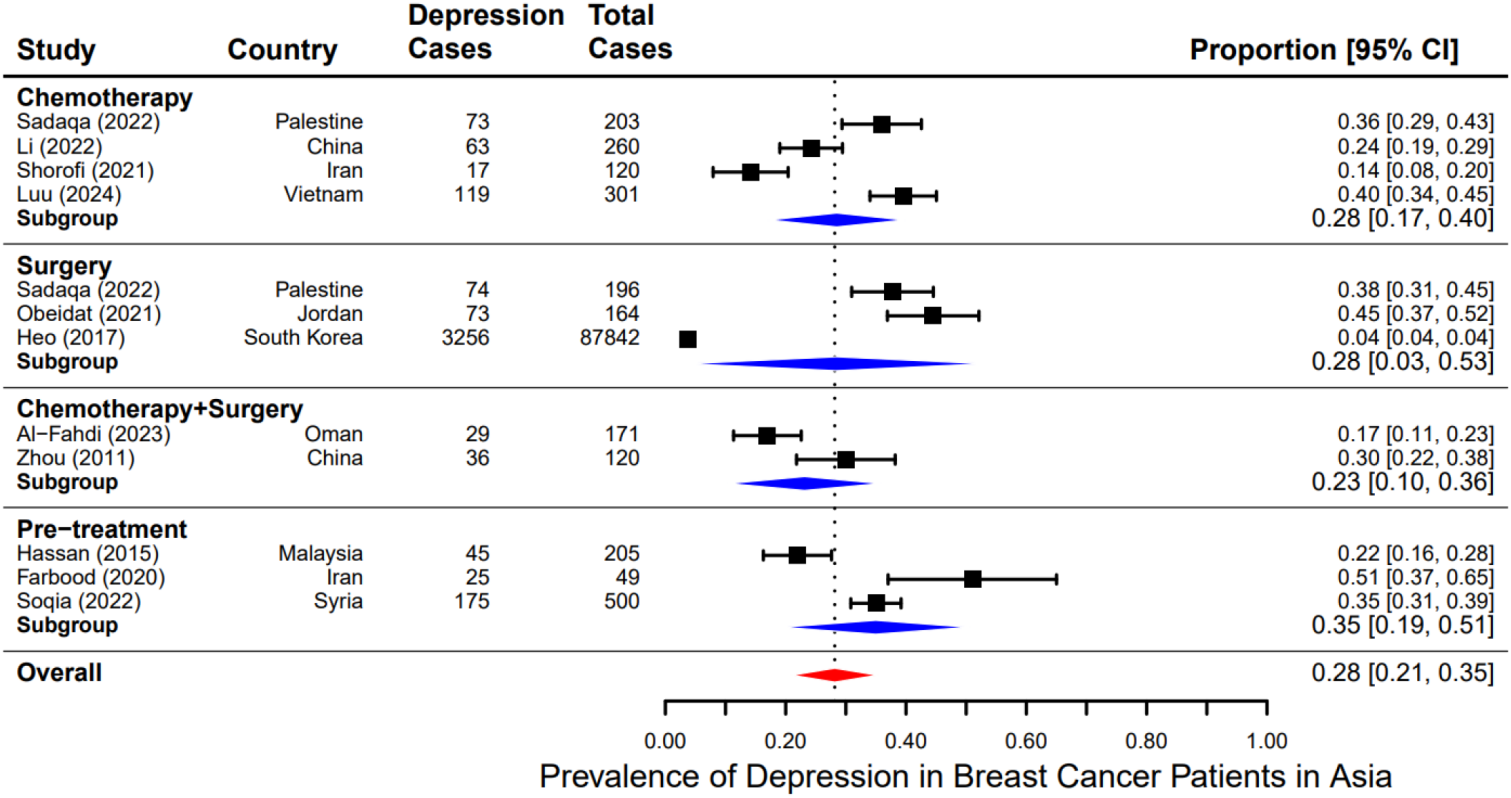
Subgroup analysis of pooled prevalence of depression among breast cancer patients by type of study participants.

Additionally, one subtype analysis was performed to examine the impact of treatment modalities (chemotherapy, surgery, both, and no treatment) on depression rate. The pooled prevalence of depression was 28% (95% CI: 17%-40%) among patients receiving chemotherapy (12, 14, 24, 26), also 28% (95% CI: 3%-53%) for those underwent surgery (10, 11, 26), and was a little lower, 23% (95% CI: 10%-36%) (21, 22), in patients who had received both chemotherapy and surgery. Patients who had not undergone treatment showed a slightly higher prevalence of 35% (95% CI: 21%-35%) (13, 23, 25). However, no statistically significant differences were detected across these subgroups (p > 0.05) (**Figure 4**), though these analyses had limited power due to the small number of studies in each category.

### Sensitivity analysis

**Figure 5:**
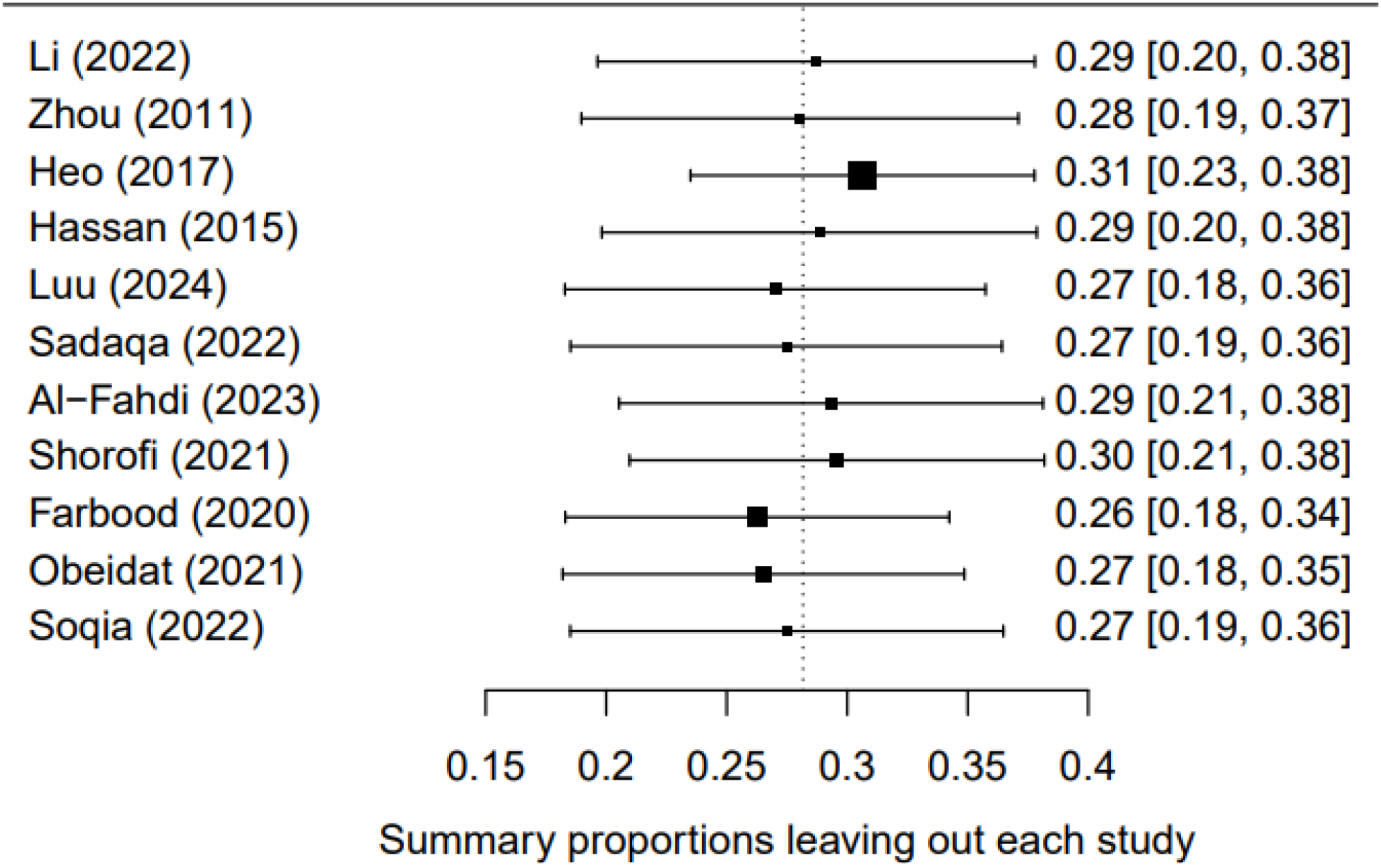
Sensitivity analysis of prevalence of depression among breast cancer patients in Asia.

To evaluate the robustness of the study findings, we conducted a sensitivity analysis using the leave-one-out method, wherein each study was sequentially excluded and the pooled effect size was recalculated. In all pooled results after exclusion, the effect sizes remained consistent, indicating that no single study had a significant impact on the overall results (**Figure 5**).

### Publication bias detection

**Figure 6:**
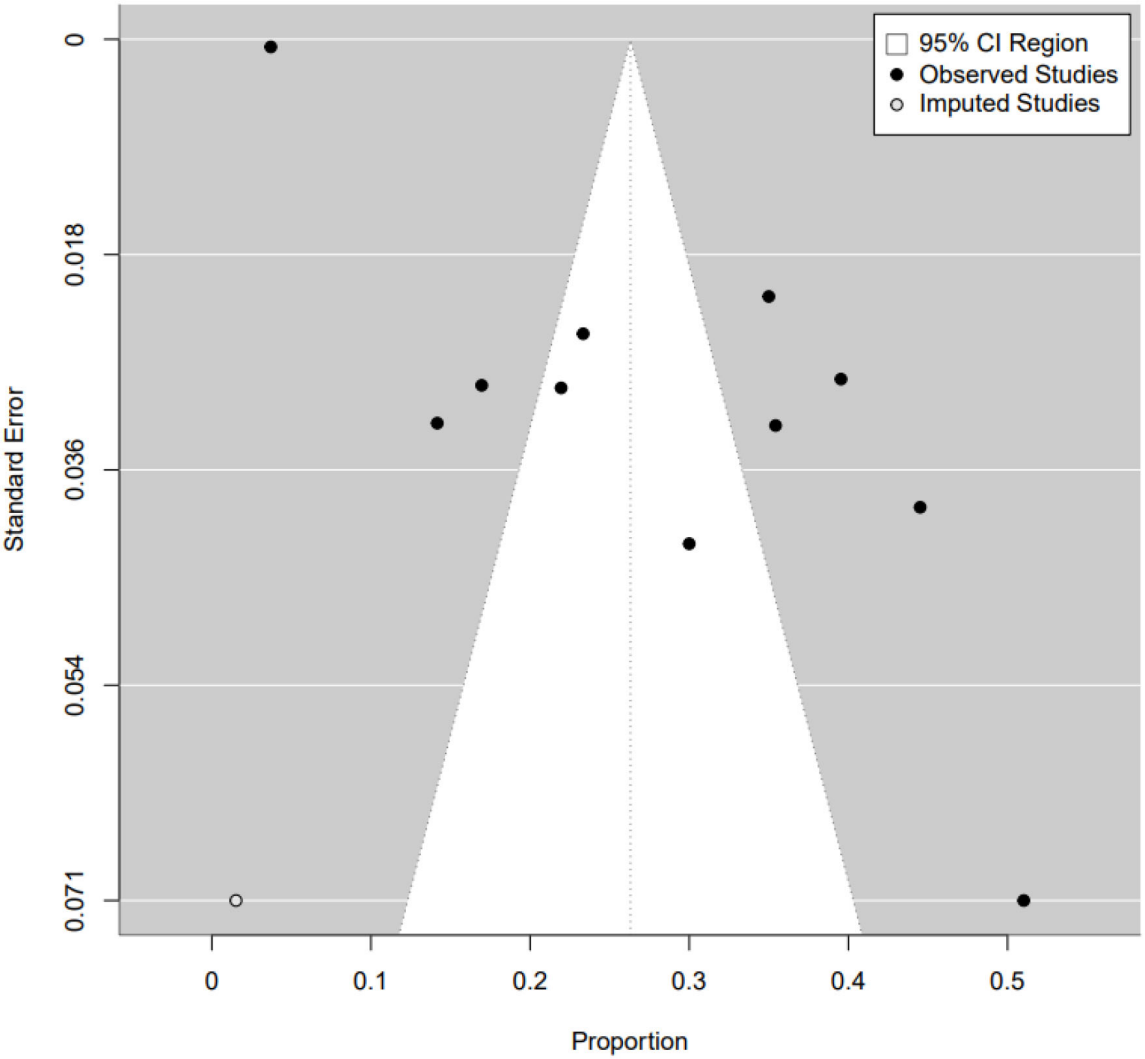
Publication bias of prevalence of depression among breast cancer patients in Asia.

We also assessed the potential for publication bias using funnel plots and Egger’s test. Visual inspection of the funnel plot did not reveal significant asymmetry, but Egger’s test argues against this with a p-value of 0.0018. To further investigate, we conducted a trim-and-fill analysis, which indicated that the overall results remained similar to the original pooled results after accounting for potentially unpublished literature. Therefore, the unpublished literature did not affect the conclusions of this study, demonstrating the robustness of the findings (**Figure 6**).

## Discussion

Based on the present findings, the pooled prevalence of depression among breast cancer patients in Asia was 28%. This indicates that depression constitutes a significant and often underrecognized mental health burden in this population and should be integrated as a crucial component of comprehensive cancer care. However, subgroup analyses revealed differences in pooled prevalence rates across countries and treatment modalities, though these were not statistically significant and should be considered inconclusive due to the limited number of studies within each subgroup. This may be attributed to the limited number of relevant studies, resulting in insufficient statistical power to detect significant differences. On the other hand, it also suggests that, beyond geographical region and treatment modality, more complex socioeconomic and cultural factors collectively influence the risk of depression in breast cancer patients. Future research should consider examining patient coping mechanisms (27–29), psychosocial support systems (30–32), and economic factors (18, 29), which may play more prominent roles in patients’ psychological well-being. Furthermore, it is crucial to note the extreme heterogeneity across studies (I² = 99%). The reported prevalence ranged from 3.7% to 51%, and the 95% prediction interval was very wide. Consequently, the single pooled point estimate (28%) offers limited insight for individual patients across diverse Asian settings. Additionally, although the trim-and-fill analysis suggests robustness, the significant Egger’s test indicates that our pooled estimate may be modestly inflated—a common challenge in depression prevalence research. Therefore, interpretation should prioritize the observed range and prediction interval over the point estimate.

This study found that the pooled prevalence of depression in breast cancer patients is lower than previously reported global rates (32.2%) (17) and rates in sub-Saharan Africa (45.6%) (33), but higher than those reported in the Americas (25.1%) and Europe (27.2%) (17). These disparities may primarily stem from differences in healthcare expenditure, socio-cultural attitudes (27–29), economic status (18, 29), and accessibility of healthcare services (30–32). In regions with well-funded and accessible healthcare systems, such as South Korea (10), the prevalence of depression was notably lower (4%). It is important to note that the study by Heo et al. relied on ICD codes to identify depression, which likely captures only diagnosed or treated cases, thereby underestimating the true burden. In contrast, screening scales are more sensitive. Therefore, this low prevalence is more likely attributable to methodological differences rather than a true regional variation. This lower rate may also benefit from easier access to mental healthcare, earlier diagnosis, and more robust support systems. Consistent with this hypothesis, depression prevalence was higher in countries with lower healthcare expenditure and underdeveloped infrastructure, such as parts of West Asia (11, 25). Furthermore, socio-cultural attitudes towards mental health vary greatly across Asia, influencing the recognition and reporting of depressive symptoms. For instance, a longitudinal study among Chinese breast cancer patients indicated a significant prevalence of fear of cancer recurrence, which was positively associated with concurrent symptoms of depression and anxiety (29). This suggests that psychological distress, including depressive symptoms, is a salient and recognized experience in this cultural context, potentially influencing how distress is expressed and reported in clinical settings. In some cultural contexts, patients may tend to express psychological distress through somatic symptoms rather than emotional distress, leading to under-recognition of depression.

In low-income regions, breast cancer patients often face a heavy economic burden, which not only affects their treatment outcomes but also significantly increases their risk of developing depression (23). Breast cancer treatment typically requires long-term medical interventions, including surgery, radiotherapy, chemotherapy, and prolonged medication, all of which can be prohibitively expensive in low-income countries (34, 35). Due to limited healthcare expenditure and underdeveloped public health infrastructure in these regions, many patients struggle to access necessary treatments, leading to disease progression and exacerbated psychological distress (36, 37). Additionally, patients in low-income areas often bear high costs related to transportation and accommodation, further intensifying their financial pressure (17, 31). The lack of a robust social security system prevents patients from receiving adequate financial support, which is another reason for the observed higher prevalence of depression in these populations.

To address these challenges, governments and non-governmental organizations must collaborate to establish and expand financial assistance programs to help breast cancer patients cover treatment costs. Strengthening primary healthcare systems to improve early diagnosis and treatment accessibility is also crucial, as this can reduce the overall cost burden associated with late-stage cancer treatment (38). In resource-limited settings, priority can be given to low-cost, scalable interventions, such as psychological screening, brief psychological interventions, peer support programs, or community-based support networks implemented by primary healthcare workers. Furthermore, increasing public awareness and education about breast cancer and its psychological impact can help reduce the stigma associated with the disease and mental health issues. Establishing patient support groups and providing psychological counseling services are essential for offering the emotional support patients need to cope with the challenges of the disease. Secondly, psycho-oncology services have not been systematically integrated into standard oncology care pathways in many Asian countries. The shortage of dedicated mental health professionals and unclear referral pathways may further limit the identification and intervention of depression in breast cancer patients. In summary, comprehensive intervention strategies targeting the economic burden on breast cancer patients in low-income regions, and integrating mental health assessment and intervention throughout the breast cancer care pathway (e.g., at diagnosis, during treatment, and at follow-up), are crucial for achieving a patient-centered comprehensive oncology care model and for improving their quality of life and mental health outcomes.

From a clinical practice perspective, the findings of this study suggest that the focus of integrating psychological care lies not merely in whether depression screening is conducted, but in *how* it is conducted and the subsequent linkage to care pathways. Given the limited and uneven distribution of mental health resources in many Asian countries, relying solely on referrals to psychiatric specialists may be impractical. In contrast, embedding brief psychosocial assessment tools into routine oncology workflows (e.g., at initial diagnosis, treatment transition points, or follow-up) and training oncology nurses or primary healthcare workers to recognize psychological distress may be a more feasible strategy. Particularly in regions with heavy economic burdens or fragmented support systems, ensuring that positive screening results are linked to accessible support resources (such as peer support, basic psychological interventions, or community-based projects) may improve patient mental health outcomes more effectively than simply increasing the screening rate.

**Figure 7:**
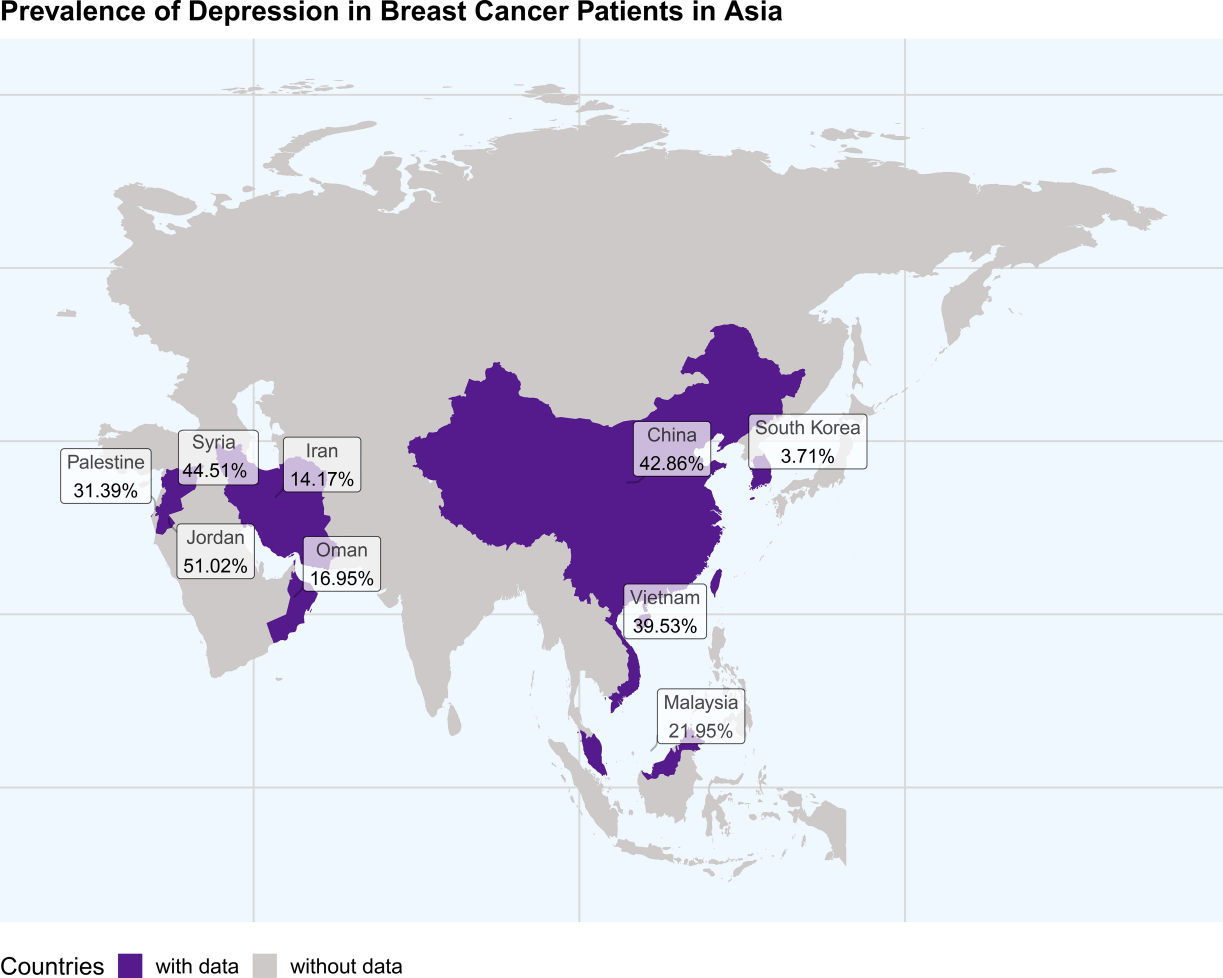
Prevalence map of depression in breast cancer patients in Asia.

Although this study is one of the earliest systematic assessments of the prevalence of depression among breast cancer patients in Asia, it is not without limitations. First, our search strategy was restricted to literature in English and Chinese, which may have led to the omission of relevant studies published in other local Asian languages (e.g., Korean, Japanese, Arabic), thereby introducing language bias. Considering that significant research findings in resource-limited settings are often published in local languages, this bias may create uncertainty in our overall prevalence estimate. Second, the sample size was relatively limited, and the geographical distribution was uneven, particularly due to a lack of data from countries with less developed healthcare systems (**Figure 7**). This limitation may restrict the generalizability of our findings to the broader Asian context. Furthermore, the sample was primarily drawn from countries with more advanced medical infrastructure, which may have led to an overestimation of the prevalence, as these countries typically offer more extensive mental health support services. Moreover, this study did not account for breast cancer stage. Given the established link between disease severity and mental health outcomes, stage likely significantly influences depression prevalence. Additionally, due to missing data, we were unable to perform subgroup analyses based on age, income level, or education level, which limits our ability to fully explore the impact of these socioeconomic factors on depression prevalence. Future research should consider including larger, more homogeneous samples within each treatment category and explore other factors that may more profoundly influence depression outcomes, such as socioeconomic status, mental health service accessibility, and cultural differences. Furthermore, based on the relatively high prevalence of depression found in this study, it is recommended to implement routine depression screening for breast cancer patients in Asia. Priority can be given to tools already validated in Asian populations, such as the PHQ-9, HADS, or CES-D, using culturally validated cut-off values to improve the accuracy of depression identification.

## Data Availability

The original contributions presented in the study are included in the article/supplementary material. Further inquiries can be directed to the corresponding author.
